# Sex-Specific Metabolic Impairments in a Mouse Model of Disrupted Selenium Utilization

**DOI:** 10.3389/fnut.2021.682700

**Published:** 2021-05-10

**Authors:** Penny M. Kremer, Daniel J. Torres, Ann C. Hashimoto, Marla J. Berry

**Affiliations:** ^1^Department of Cell and Molecular Biology, John A. Burns School of Medicine, University of Hawaii at Manoa, Honolulu, HI, United States; ^2^Pacific Biosciences Research Center, University of Hawaii at Manoa, School of Ocean and Earth Science and Technology, Honolulu, HI, United States

**Keywords:** selenium, selenoproteins, sex differences, selenocysteine lyase, metabolic syndrome

## Abstract

The essential micronutrient selenium (Se) provides antioxidant defense and supports numerous biological functions. Obtained through dietary intake, Se is incorporated into selenoproteins *via* the amino acid, selenocysteine (Sec). Mice with genetic deletion of the Se carrier, selenoprotein P (SELENOP), and the Se recycling enzyme selenocysteine lyase (SCLY), suffer from sexually dimorphic neurological deficits and require Se supplementation for viability. These impairments are more pronounced in males and are exacerbated by dietary Se restriction. We report here that, by 10 weeks of age, female *Selenop*/*Scly* double knockout (DKO) mice supplemented with 1 mg/ml sodium selenite in drinking water develop signs of hyper-adiposity not seen in male DKO mice. Unexpectedly, this metabolic phenotype can be reversed by removing Se from the drinking water at post-natal day 22, just prior to puberty. Restricting access to Se at this age prevents excess body weight gain and restriction from either post-natal day 22 or 37 reduces gonadal fat deposits. These results provide new insight into the sex-dependent relationship between Se and metabolic homeostasis.

## Introduction

Selenium (Se) has been implicated in a wide range of biological functions that are critical for human health (1). This essential trace element is translationally incorporated into selenoproteins, as the amino acid, selenocysteine (Sec). Selenoproteins, in turn, comprise a major component of the antioxidant defense system of many different tissues. Se is acquired *via* dietary intake and utilized in particularly high levels by the liver, kidneys, brain, testes, and skeletal muscle (2). Distribution of Se throughout the body requires the combined actions of the Se carrier, Selenoprotein P (SELENOP) and the Se recycling enzyme selenocysteine lyase (SCLY). Following absorption by the gut and transport to the liver *via* the portal vein, Se is used to synthesize SELENOP, which contains multiple Sec residues. After being secreted into the bloodstream, SELENOP is taken up by target tissues to be catabolized intracellularly. Proper utilization of the delivered Sec residues is dependent on SCLY, which catalyzes the breakdown of Sec into selenide, to be used for *de novo* selenoprotein biosynthesis (3).

The role of Se in energy homeostasis is complicated, as clinical studies have correlated both Se deficiency and high Se intake with metabolic disease in humans (4). Hepatic SELENOP has been implicated in the development of hyperglycemia (5) and insulin resistance (6) in humans and mice, respectively. Mice with genetic knockout (KO) of *Scly* have increased susceptibility to metabolic syndrome (7) and diet-induced obesity (8), with more dramatic effects observed in male mice. Additionally, targeted deletion of specific selenoproteins causes differential metabolic disturbances in animal models (9–11), demonstrating the impact of not only dietary Se intake, but also proper Se utilization.

We previously bred *Scly* KO and *Selenop* KO mouse strains to produce double knockout (DKO) mice. DKO mice were found to suffer from severe neurological dysfunction (12), which was subsequently found to be attenuated by prepubescent castration (13). We recently reported that although female DKO mice display less severe neurological deficits than their male counterparts, the phenotype is worsened by the removal of Se supplementation during puberty (14). Here we provide preliminary data showing that female DKO mice exhibit a metabolic phenotype not seen in male DKO mice.

## Materials and Methods

The data in this report were generated from mice used in our previous publication addressing the sex-specific neurological phenotype of DKO mice (14). Male and female C57/BL6N wild-type (WT) and DKO mice were generated as previously described [9]. Since supplementation with Se is critical for DKO mouse survival, all subjects in this study were maintained on standard mouse chow containing ~0.25 ppm Se and drinking water containing 1 mg/ml sodium selenite, Na_2_SeO_3_. Mice were given *ad libitum* access to food and water from weaning (~18 days of age) until the age of 10 weeks, at which point they were weighed, sacrificed *via* CO_2_ asphyxiation and tissues were harvested. Inguinal and gonadal white adipose tissue (WAT) deposits were removed and weighed on a benchtop analytical balance. For some groups, Se-supplemented drinking water was replaced with non-supplemented drinking water at either 22 days or 37 days post-natal (denoted as -NoSeP22 and -NoSeP37, respectively). All procedures and experimental protocols involving animals were approved by the University of Hawaii's Institutional Animal Care and Use Committee. Animal Care and Use Committee (IACUC) Protocol: “Mechanism of Selenoprotein Synthesis and Studies of Selenoprotein Functions:” APN 09-871-9, approved: 18 July 2019. Institutional Biosafety Committee (IBC) Protocol: “Mechanism of Selenoprotein Synthesis and Studies of Selenoprotein Functions:” IBC #18-10-544-02-4A-1R, approved: 23 October 2018.

## Results

**Figure 1:**
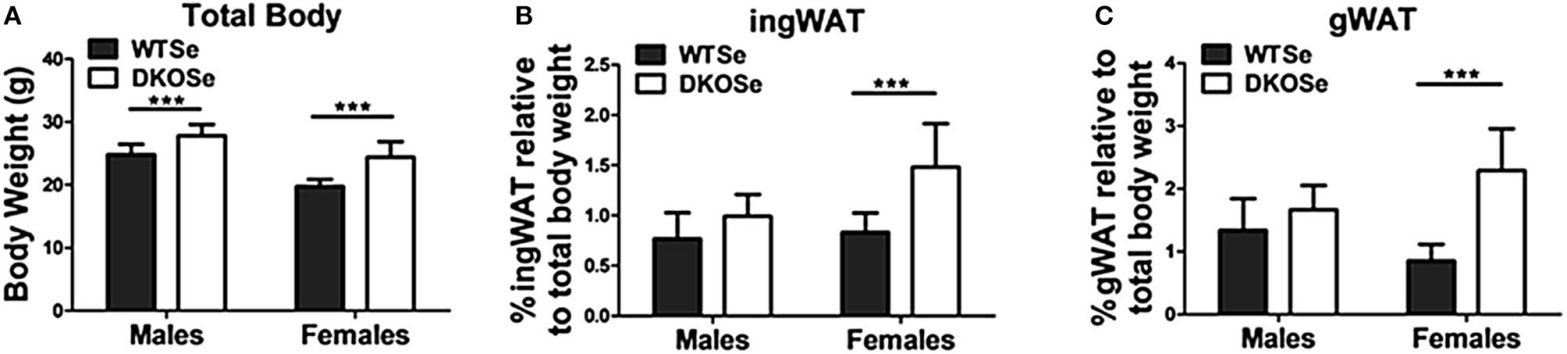
Body composition of wild-type (WT) and double knockout (DKO) mice of both sexes. **(A)** Total body weight, **(B)** percentage of inguinal white adipose tissue (ingWAT), and **(C)** percentage of gonadal white adipose tissue (gWAT) weight of male and female WT and DKO mice at 10 weeks of age. Two-way ANOVA: Total body weight Interaction NS, Genotype *F*_(1,52)_ = 60.68, *p* = <0.0001, Sex *F*_(1,52)_ = 75.15, *p* < 0.0001; ingWAT Interaction *F*_(1,52)_ = 7.54, *p* = 0.0083, Genotype *F*_(1, 52)_ = 31.51, *p* < 0.0001, Sex *F*_(1,52)_ = 12.64 *p* = 0.0008; and gWAT Interaction *F*_(1,52)_ = 18.64, *p* < 0.0001, Genotype *F*_(1,52)_ = 47.51, *p* < 0.0001, Sex NS; *n* = 14 all groups. Bonferroni's multiple comparisons test: ***p < 0.001. All values are reported as mean ± SEM.

Female DKO mice had significantly higher total body weights compared to their WT counterparts at 10 weeks of age (Figure 1A). WAT deposits were also significantly heavier in female DKO mice compared to WT controls (Figures 1B,C), an effect that was not observed in male DKOSe mice. These results indicate a metabolic effect specific to female DKO mice.

**Figure 2:**
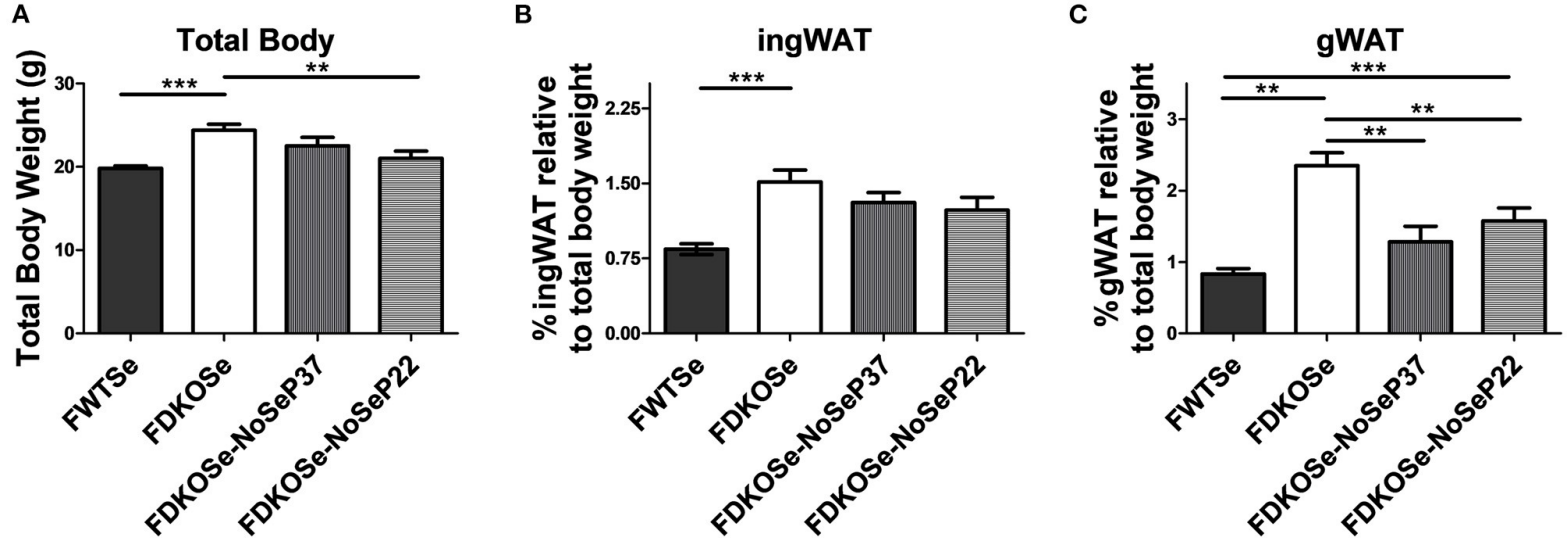
Effect of dietary Se removal on the body composition of female double knockout (DKO) mice. **(A)** Total body weight, **(B)** percentage of ingWAT, and **(C)** percentage of gWAT weight at 10 weeks of age of female wild-type (FWT), female double-knockout (FDKO), and female double knockout with Se supplementation removed at post-natal day 37 (FDKOSe-NoSeP37) or post-natal day 22 (FDKOSe-NoSeP22). One-way ANOVA: Total body weight *F*_(3,39)_ = 8.46, *p* = 0.0002; %ingWAT *F*_(3,39)_ = 7.29, *p* = 0.0005; %gWAT *F*_(3,39)_ = 17.08, *p* < 0.0001; *n* = 13 all groups except P37 *n* = 4; Bonferroni's multiple comparisons test: **p < 0.01, ***p < 0.001. All values are reported as mean ± SEM.

Previously, we demonstrated that removal of Se supplementation from female DKO mice prior to puberty at post-natal day 22 (P22) exacerbates the neurological phenotype to a similar level as male DKO mice without Se removal (14). We report here that, surprisingly, total body weight was significantly lower in female DKO when Se water was removed at P22 compared to female DKO mice with constant Se supplementation (Figure 2A). Regarding fat deposits, inguinal WAT weights trended lower in female DKO mice when Se was removed at P22 (Figure 2B). Removal of Se supplementation at either P22 or P37 significantly reduced gonadal WAT deposits in female DKO mice (Figure 2C). These data demonstrate that while female DKO mice develop a metabolic phenotype not seen in male DKO mice, this phenotype is partially attenuated by the removal of Se-supplemented drinking water.

## Discussion

Although concurrent KO of S*elenop* and *Scly* in female mice causes a milder neurological phenotype compared to male DKO mice, we report here that female DKO exhibit an obesogenic phenotype marked by elevated WAT deposit weights. These results are consistent with past studies showing that *Scly* KO mice are predisposed to similar metabolic deficits (7, 8). This predisposition is more pronounced in male *Scly* KO mice, which contrasts with our current findings that female DKO mice exhibit hyper-adiposity while males do not. Considering that male DKO mice suffer from severe motor deficits and seizures, even while supplemented with Se, however, it is possible that these symptoms could mask a metabolic phenotype by affecting their ability to ambulate and eat. Thus, further characterization of DKO mice should involve analysis of physical activity and feeding behavior. Future studies should also evaluate core temperature, respiratory metabolism, and a more comprehensive analysis of adiposity, as Se has been shown to regulate adipose tissue thermogenesis (15) and lipid metabolism (16). Finally, a broad assessment of circulating hormones, such as plasma insulin, leptin, and thyroid hormones, as well as nutrients, such as glucose, triglycerides, and free fatty acids, would help detect changes in the endocrine regulation of body fat stores.

**Figure 3:**
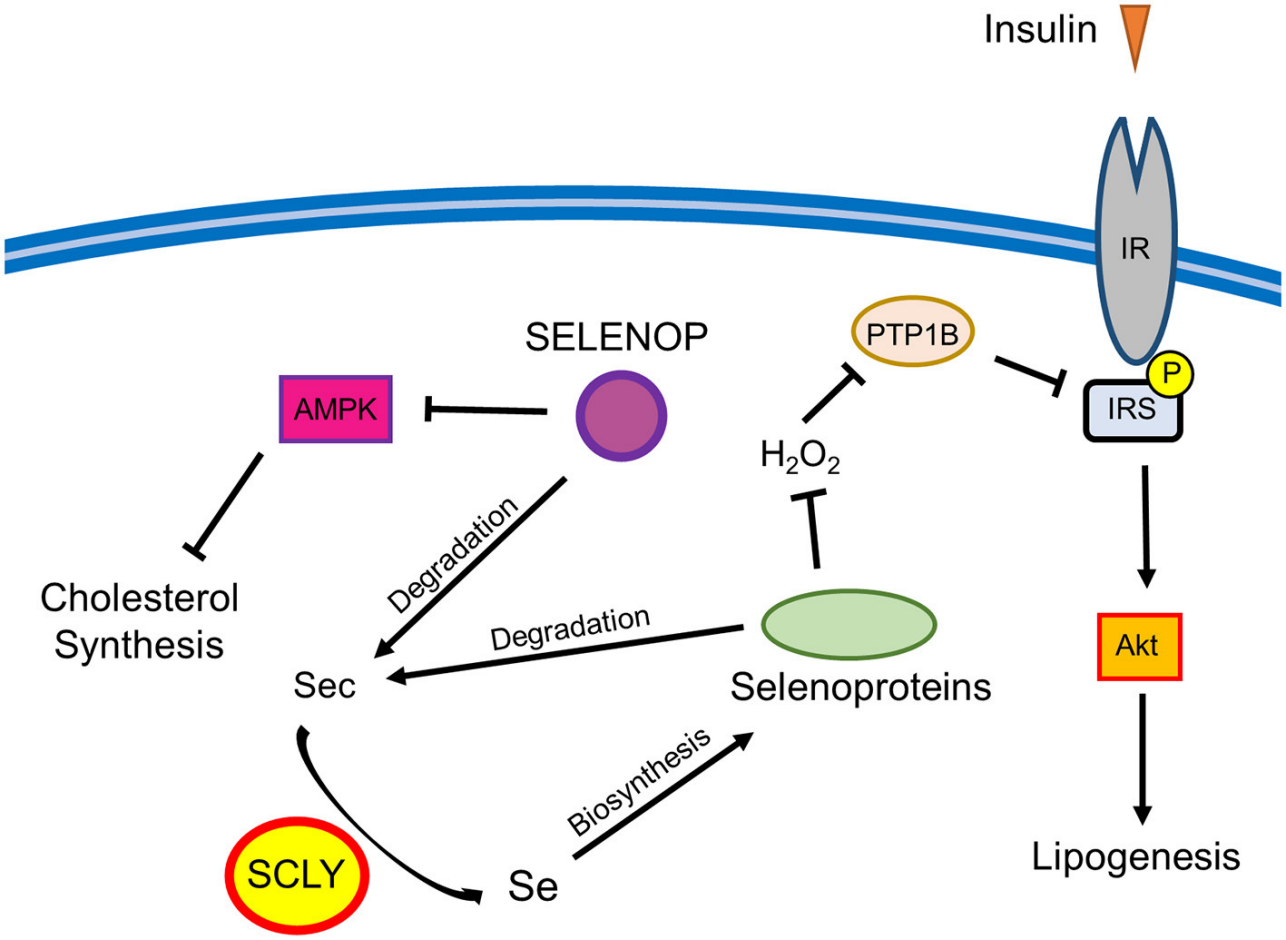
Schematic representation of the possible interactions between Se metabolism and lipid metabolism. Selenoprotein P (SELENOP) has shown an ability to de-activate AMP-activated protein kinase (AMPK) and inhibit insulin signaling in liver and skeletal muscle (6). AMPK limits cholesterol synthesis in the liver, thus representing a potential node through which SELENOP may regulate cholesterol. SELENOP and other selenoproteins can be degraded to produce selenocysteine (Sec) residues that are further metabolized through a process involving selenocysteine lyase (SCLY) to produce Se. This Se can then be used for *de-novo* synthesis of selenoproteins. The antioxidant activity of selenoproteins may promote the negative regulation of insulin signaling by preventing the oxidative inactivation of protein tyrosine phosphatase 1B (PTP1B) by hydrogen peroxide (H_2_O_2_). PTP1B de-phosphorylates the IR and insulin receptor substrate (IRS), which, in turn, reduces protein kinase B (AKT)-mediated lipogenesis. Through affecting this pathway, SCLY could work to limit lipogenesis (7).

Surprisingly, challenging female DKO mice with the removal of Se supplementation in drinking water partially prevented the development of excess weight gain and hyper-adiposity. These results are in contrast with our previous findings that *Scly* KO mice develop signs of metabolic syndrome, including obesity, when raised on a Se-deficient diet (7). This implies that the added effect of *Selenop* deletion, which limits the ability of the body to distribute Se, alters the metabolic response to dietary Se restriction in mice lacking *Scly*. It is possible that the baseline redox environment in DKO mice is dramatically different from *Scly* KO mice, thus altering the compensatory mechanisms implemented in response to changes in antioxidant availability. These changes are likely complex as Se and selenoproteins have shown a capacity to differentially regulate energy metabolism through a variety of physiological processes, such as thyroid hormone metabolism (17) and insulin activity (18), as well as tissues that closely regulate energy metabolism including liver (19), pancreas (20), and the hypothalamus (21). This phenomenon is somewhat reminiscent of the observation that there appears to be a relatively narrow range of Se intake that is beneficial in humans as both low and high levels of Se status have been connected to an increased risk for type 2 diabetes (4). Thus, it is possible that the beneficial window is somehow lowered in female mice under the conditions of S*elenop*/*Scly* double KO, and that the combination of Se intake from both food and water surpasses the upper limit of that window. This could possibly explain why, in regard to metabolic phenotype, removal of Se water, while maintaining some Se intake *via* food consumption, appears to have a beneficial effect on female DKO mice. There are multiple pathways through which Se may affect adiposity by regulating lipid metabolism, including cholesterol synthesis and insulin signaling (7). For example, SCLY-mediated selenoprotein activity may negatively regulate the ability of insulin to induce lipogenesis in the liver by promoting protein tyrosine phosphatase 1B (PTP1B) antagonism of insulin signaling. On the other hand, SELENOP may regulate glucose metabolism in both the liver and skeletal muscle by actin on AMP-activated protein kinase (AMPK) (6). Thus, the deletion of both the *Selenop* and *Scly* genes may disrupt multiple pathways affecting adiposity. The possible intersection between Se metabolism and lipid metabolism is described in Figure 3.

On a final note, it is possible that the reduction of the female DKO mouse hyper-adiposity phenotype brought on by Se water removal may be affected by worsening neurological symptoms (14). It is important to note, however, that while gonadal WAT deposits were reduced in female DKO mice by Se removal at either P22 (just prior to puberty) or P37 (latter stages of puberty), the neurological phenotype of female DKO mice was shown to be aggravated only by Se removal at P22, not at P37 (14). Since female DKO mice with Se removed at P37 show no changes in neuromotor function as a result, it is, thus, likely that the reduced gonadal WAT deposits result from a distinct mechanism central to energy homeostasis. Taken together, these data implicate S*elenop*/*Scly* DKO mice as a useful model for investigation of these relationships and warrant comprehensive metabolic characterization of these mice and interrogation of underlying mechanisms.

## Data Availability Statement

The raw data supporting the conclusions of this article will be made available by the authors, without undue reservation.

## Ethics Statement

The animal study was reviewed and approved by University of Hawaii's Institutional Animal Care and Use Committee.

## Author Contributions

PK, AH, and MB were involved in conceptualization, experimental design, obtaining resources, and interpretation of data. PK and AH were involved in data acquisition and analysis. PK was involved in writing the initial drafts. DT was involved in data validation, interpretation of data, writing the initial drafts, and manuscript revisions. MB was involved in manuscript revisions and providing funding. All authors read and approved of the final manuscript.

## Conflict of Interest

The authors declare that the research was conducted in the absence of any commercial or financial relationships that could be construed as a potential conflict of interest.
